# Physical activity, weight management, and mental health during COVID-19 lockdown: A cross-sectional study of healthcare students in China

**DOI:** 10.1371/journal.pone.0302894

**Published:** 2024-10-17

**Authors:** Rong Zhang, Yuhuan Yin, Yiyin Zhang, Yuping Feng, Hongyan Meng, Jing Wang, Min Zhang, Juxia Zhang

**Affiliations:** 1 School of Nursing, Gansu University of Chinese Medicine, Lanzhou, Gansu, China; 2 Department of Thoracic Surgery, The Second Affiliated Hospital of Air Force Medical University, Xi’an, Shaanxi, China; 3 Scientific Research Office, Gansu Provincial Hospital, Lanzhou, Gansu, China; 4 Clinical Educational Department, Gansu Provincial Hospital, Lanzhou, Gansu, China; Khyber Medical University, PAKISTAN

## Abstract

**Background:**

There is growing evidence that the social blockade brought about by the COVID-19 pandemic has dramatically affected college students’ physical activity; however, their weight management behaviors and mental health have not been fully explored, especially among healthcare students. This study aimed to assess physical activity, weight management behaviors, and mental health among healthcare students during the campus lockdown and to analyze the factors influencing physical activity.

**Methods:**

A cross-sectional survey of 1,216 healthcare students from March 24, 2022, to April 11, 2022. To collect information about students’ physical activity, weight management behaviors, and mental health, they were asked to complete the Physical Activity Questionnaire-Short Form (IPAQ-SF), 11 issues related to weight management behavior, and the World Health Organization 5-Item Well-Being Index (WHO-5) questionnaire. Binary logistic regression was performed to determine the factors influencing physical activity.

**Results:**

Almost half of the participants had low levels of physical activity (45%), the mean score for weight management behavior was 39.55±6.26, and 54.6% had low levels of well-being. Healthcare students without exercise habits during the non-epidemic period reported lower levels of physical activity (OR = 1.81; 95%CI = 1.41–2.34, *P*<0.001). Participants with poor weight management behavior were more likely than other participants to report lower levels of physical activity (OR = 0.92; 95%CI = 0.90–0.94, *P* < 0.001). Likewise, the odds of being physically inactive were higher among the happier participants (OR = 0.98; 95%CI = 0.96–0.99, *P* = 0.031).

**Conclusions:**

During the COVID-19 lockdown, most healthcare students in Gansu province lack physical exercise and have a low health level. Significant correlation factors for low physical activity levels were grade level, non-lockout exercise habits, weight management level, and well-being. These findings demonstrate the importance of developing targeted policies and programs encouraging physical activity among medical students.

## Introduction

The novel coronavirus (COVID-19) pandemic began in December 2019 and spread rapidly in a short time, causing severe effects in many countries worldwide [1]. In response to the epidemic, several governments, particularly the Chinese, have implemented "partial lockdown" and "social isolation" strategies, closing schools, factories, and other public places. These strategies have effectively reduced cross-contamination [2].

While these restrictions have been effective in curbing the spread of the epidemic, this extraordinary social experience has also had many negative effects on people’s mental health [3, 4], with the education and healthcare industries particularly affected [5]. The social lockdown triggered by COVID-19 has dramatically changed students’ living and learning styles, affecting their physical and mental health and increasing the incidence of depression and anxiety [6, 7]. The pandemic may also be particularly harmful to the well-being of young people [8]. Like other students, healthcare students have experienced disruption in education, loss of peer support networks, and uncertainty about volunteering in hospitals during the pandemic [5]. Studies have found that mental health problems are on the rise among students majoring in healthcare [9, 10], and mental health problems are more common among students majoring in health care than students from other majors [11].

Physical activity and regular exercise are essential for promoting an active and healthy lifestyle [12]. Research has shown that regular physical activity improves college students’ mental health and well-being [13]. The World Health Organization (WHO) recommends at least 150 minutes of moderate physical activity, 75 minutes of vigorous exercise, or a combination of both every week [14]. However, due to the COVID-19 outbreak, daily physical activity and outdoor sports were limited [15–17]. Sedentary behavior increased significantly, which was the most serious among young people. A Chinese study showed that during the COVID-19 outbreak, about 52.3% of Chinese college students had insufficient physical activity [6]. The total time required for medical students to acquire the necessary professional knowledge and skills is longer than for students in other majors, and they need to spend more time on their courses [9]. Before the crisis, a study found that many of the nursing and medical students’ physical activity did not reach the level of advice [18], and lower levels of physical activity among healthcare students may have been exacerbated by the COVID-19 pandemic and subsequent lockdown [19].

In addition, weight management may struggle during the COVID-19 pandemic. A survey of U.S. adults found that 37.2% of participants reported gaining weight during the outbreak lockdown [20]. Similarly, a study of 1013 youth in Malaysia found that 48.6% had gained weight due to the epidemic embargo [21]. Increased sedentary behavior, decreased physical activity, increased frequency of snacking (especially after dinner), increased alcohol intake, reduced water intake, emotional eating, decreased sleep quality, and overweight/obesity were identified as risk factors for weight gain [22]. Many students developed unhealthy mechanisms during the pandemic to cope with their heightened feelings of anxiety, stress, or depression [23]. Due to the longer course length, medical students are likelier to have unhealthy lifestyles [24]. A survey of nursing students found that almost half of them gained weight and did not exercise regularly [25]. There are still relatively few investigations of medical students’ weight management behaviors during the lockdown, and more attention should be given to this population.

Healthcare students will take on frontline healthcare provider roles in the future, and their role in the design and delivery of healthcare is critical. However, to the best of our knowledge, there is still little research on the impact of COVID-19 on the mental health, physical activity, and weight management of healthcare students in China. This study aimed to examine the effects of social isolation during COVID-19 on PA levels, weight management behaviors, and well-being in healthcare students and to explore their correlation. We hypothesized that students would have decreased PA levels, reduced active weight management behaviors, and lower levels of well-being. The findings will help to understand the negative changes in healthcare students during the COVID-19 crisis and will be critical to better planning support programs for healthcare students.

## Methods

### Sample and study design

This is a cross-sectional study of healthcare students. The data were collected from March 24, 2022, to April 11, 2022. During the data collection period, social blockade restrictions remained, including online teaching, classroom teaching, physical education, unnecessary school holidays, meeting with other family members, and other gatherings. Our data came from six universities with medical-related programs in Gansu Province that took measures to close their schools in March-April 2022 when the COVID-19 outbreak returned. The four universities in Lanzhou City, Gansu Province, include Gansu University of Traditional Chinese Medicine, Lanzhou University, Northwest Minzu University, and Gansu Health Vocational College. Gansu Medical College is located in Pingliang City, Gansu Province. Hexi University is located in Zhangye City, Gansu Province. Our majors include clinical medicine, nursing, traditional Chinese medicine, and public health. We used convenience sampling and snowball sampling. Inclusion criteria were: 18 or older, healthcare students staying at school during the COVID-19 social lockdown, and willing to participate in the survey. An online questionnaire survey was conducted on the Questionnaire Star survey platform (https://www.wjx.cn/), an online questionnaire distribution and data collection system widely used in China. The survey was conducted using an anonymous, self-administered questionnaire, and a pilot survey was conducted prior to the inspection. 1224 questionnaires were collected, and 1216 effective questionnaires were obtained, with an effective rate of 99.3%.

### Measures

The online survey consists of four parts: (1) Socio-Demographic Characteristics; (2) Physical activity; (3) Weight management behavior; (4) Mental health.

### Socio-demographic characteristics

Demographic data include gender, age, Annual household income, Education background, Academic year, Majors, BMI (according to Chinese population guidelines: <18.5, underweight; 18.5–24.0, normal weight; >24.0, overweight; ≥28.0, obesity) [26]. To understand the exercise habits of participants during the non-COVID-19 epidemic blockade, we used the question “exercise during the non-COVID-19 blockade period (yes/ no)”.

### Physical activity

Due to the unique circumstances of the COVID-19 pandemic, most PA questionnaires for students do not apply the assessment of PA levels in schools during social isolation. We used the International Physical Activity Questionnaire-Short Form (IPAQ-SF) to assess the PA levels of college students [27]. The retest reliability of the Chinese version of the scale is 0.626 ~ 0.887, and the validity index is 0.60 ~ 0.76, which has good reliability and validity [28]. The questionnaire contains seven questions. The IPAQ-SF asked participants to recall the number of days they performed each activity (frequency) and the time they participated in each daily activity (duration) over the past seven days, as well as the average amount of time spent in sedentary behaviors [29]. According to the IPAQ-SF protocol, three distinct PA groups can be divided into three separate PA groups, considering MET/week for the sum of walking, moderate-intensity PA, and vigorous-intensity PA: low activity (<600 MET min/week); moderate activity (≥600 MET min/week) and high activity (≥3000 MET min/week) [30]. The higher the MET score, the higher the PA level in each domain [31].

### Weight management behaviors

Weight management behaviors were assessed using 11 items [31]: "Eaten a healthy and balanced diet," "Eaten large meals," "Eaten snacked," "Dieted/fasted," "Skipped meals," "Used weight control products (e.g. meal replacements)," "Exercised," "Been physically active," "Spent time sitting down," "Drank alcohol," "Got a good night’s sleep." Each question has the following answer options: A lot less, Less, A little less, The same amount, A little more, More, A lot more. Each item is rated on a seven-point scale from 1 (A lot less) to 7 (A lot more). Items 1 (eat a healthy and balanced diet), 6 (used weight control products (e.g., meal replacements), 7, (exercise), 8, (Been physical activity), 11 (Got a good night’s sleep) reverse scoring. Total score Higher values indicate poorer weight management behavior.

### Mental health

The 5-item World Health Organization Well-Being Index (WHO-5) is among the most widely used questionnaires assessing subjective psychological well-being [32]. The Chinese version of the scale has a Cronbach’s α value of 0.85 [33]. WHO-5 consists of five items: "I have felt cheerful in good spirits," "I have felt calm and relaxed," "I have felt active and vigorous," "I woke up feeling fresh and rested," and "My daily life has been filled with things that interest me." Each answer was rated on a scale rangeing from 0 (no time) to 5 (all time) [8]. A higher score on the scale represents a higher level of happiness. A total score of less than 13 indicates poor mental health and is a sign of depression [34].

### Ethics statement

The study was conducted in accordance with the Declaration of Helsinki [35]. The research proposal was approved by the research committee of Gansu Provincial Hospital (Approval Number: 2022–206). Written informed consent was obtained from all subjects and/or their legal guardians. We handled survey data confdentially and maintained anonymity of respondents throughout the study.

### Statistical analysis

All analyses were performed using SPSS software (version 22.0). Descriptive statistics include frequencies, percentages, in addition to mean ($\bar{x}$) and standard deviation (SD). Metabolic equivalent (MET) scores for walking, moderate-intensity PA (physical activity), and vigorous-intensity PA were calculated using the IPAQ scoring program. Chi-square test was used to compare differences in PA levels across demographic characteristics. Binary logistic regression was used to analyze the relationship between physical activity and general characteristics, weight management behavior and well-being. The dependent variable is the level of physical activity ((low activity (<600 MET min/week) = 0; moderate activity (≥600 MET min/week) = 1), and the independent variable is the influencing factors (general characteristics, weight management behavior, and well-being). The statistical significance was set at *p* < 0.05 (two-tailed).

## Results

### Sociodemographic characteristics

A total of 1216 participants were recruited into the study. The mean age was 21.7± 2.90 years old. Of the participants, 62.5% were female, 63.7% were normal weight. More than half of the students (54.1%) did not exercise during the non-lockdown period. The average score for weight management behavior was 39.55±6.26, and more than half of the students (54.6%) have a low level well-being (Table 1).

**Table 1:** Descriptive characteristics of the participants. (n = 1216).

		n (%)	$\bar{x}\pm \mathrm{SD}$
Gender	Male	456 (37.5)	
	Female	760 (62.5)	
Age	18–21	552(45.4)	21.7 ± 2.90
	≥22	664(54.6)	
Annual household income (¥)	<30000	558 (45.9)	
	30000–50000	352 (28.9)	
	>50000	306 (25.2)	
Education background	Undergraduate	886 (72.9)	
	Junior college student	330 (27.1)	
Major	Clinical medicine	374 (30.8)	
	Nursing	354 (29.1)	
	Public health	235 (19.3)	
	Traditional Chinese Medicine	253 (20.8)	
Academic year	First	332 (27.3)	
	Second	232 (19.1)	
	Third	232 (19.1)	
	Fourth and fifth	420 (34.5)	
Exercise (Non-lockdown period)	Yes	558 (45.9)	
	No	658 (54.1)	
BMI (kg/m^2^)	Normal	774 (63.7)	21.40 ± 3.26
	Underweight	226 (18.6)	
	Overweight or Obese	216 (17.8)	
Weight management behaviors			39.55 ± 6.26
WHO well-being score	low well-being (<13)	664 (54.6)	14.12 ± 6.00
	high well-being (>13)	552 (45.4)	

### Item scores about weight management behavior and the World Health Organization’s 5-item Well-being Index (WHO-5)

Table 2 shows that the average score of weight management behavior is 39.55 ± 6.26. Items with the highest scores for weight management behaviors: *Use of weight management products* (5.41±1.64). The lowest score on the item measuring weight management behavior: *Drink alcohol* (2.09±1.53). Highest score on the 5-item World Health Organization Well-Being Index (WHO-5): *My daily life has been filled with things that interest me* (2.99±1.44). One of the lowest-scoring entries in the WHO-5: I have felt cheerful and in good spirits (2.70 ± 1.24).

**Table 2:** Weight management behaviors and the World Health Organization’s 5-item Well-being Index (WHO-5) scores.

		Scales/subscales	$\bar{x}\pm \mathrm{SD}$
Weight management behaviors	The 3 highest score	Used weight control products (e.g. meal replacements)	5.41 ± 1.64
		Spent time sitting down	4.92 ± 1.41
		Eaten snacks	4.11 ± 1.46
	The 3 lowest score	Drank alcohol	2.09 ± 1.53
		Skipped meals	2.91 ± 1.57
		Dieted/fasted	3.01 ± 1.67
WHO well-being score	The highest score	My daily life has been filled with things that interest me	2.99 ± 1.44
	The lowest score	I have felt cheerful and in good spirits	2.70 ± 1.24

### Differences in physical activity levels by demographic characteristics

Among them, students with low activity (<600 MET min/week) accounted for 45.1%. The analysis in Table 3 showed that there were significant differences in PA activity levels among students of different ages and grades (*P* < 0.05). There was a statistically significant difference in PA activity during exercise (yes/no) during the non-blocking period (*P* < 0.001).

**Table 3:** Characteristics of university students by physical activity class.

		<600 MET-min/week n = 548(45.1), n(%)/($\bar{x}\pm \mathrm{SD}$)	≥600 MET-min/week n = 668(54.9), n(%)/($\bar{x}\pm \mathrm{SD}$)	*x* ^2^	*P*
Age				6.944	0.008*
	18–21	226(41.2)	326(48.8)		
	≥22	322(58.8)	342(51.2)		
Gender				0.174	0.721
	male	202 (36.9)	254 (38.0)		
	female	346 (63.1)	414 (62.0)		
Household income (¥)				4.608	0.100
	<30000	270 (49.3)	288 (43.1)		
	30000–50000	148 (27.0)	204 (30.5)		
	>50000	130 (23.7)	176 (26.3)		
Education background				1.930	0.174
	Undergraduate and above	410 (74.8)	476(71.3)		
	Junior college student	138(25.2)	192(28.7)		
Major				1.004	0.800
	Clinical medicine	176(32.1)	198(29.6)		
	Nursing	158(28.8)	197(29.3)		
	Public health	101(18.4)	133(20.1)		
	Traditional Chinese Medicine	113(20.6)	140(21.0)		
Academic year				10.291	0.016*
	First	128(23.4)	204(27.3)		
	Second	100(18.2)	132(19.8)		
	Third	114(20.8)	118(17.7)		
	Fourth	206(37.6)	214(32.0)		
Exercise (Non-lockdown period)				60.893	<0.001**
	Yes	184(33.6)	374(56.0)		
	No	364(66.4)	294(44.0)		
BMI (kg/m^2^)				5.549	0.062
	Normal	360(65.7)	414(62.0)		
	Underweight	86(15.7)	140(21.0)		
	Overweight or Obese	102(18.6)	114(17.1)		

Notes

* p < 0.05

** p < 0.01

### Comparison of weight management behaviors and well-being scores at different levels of physical activity

**Fig 1:**
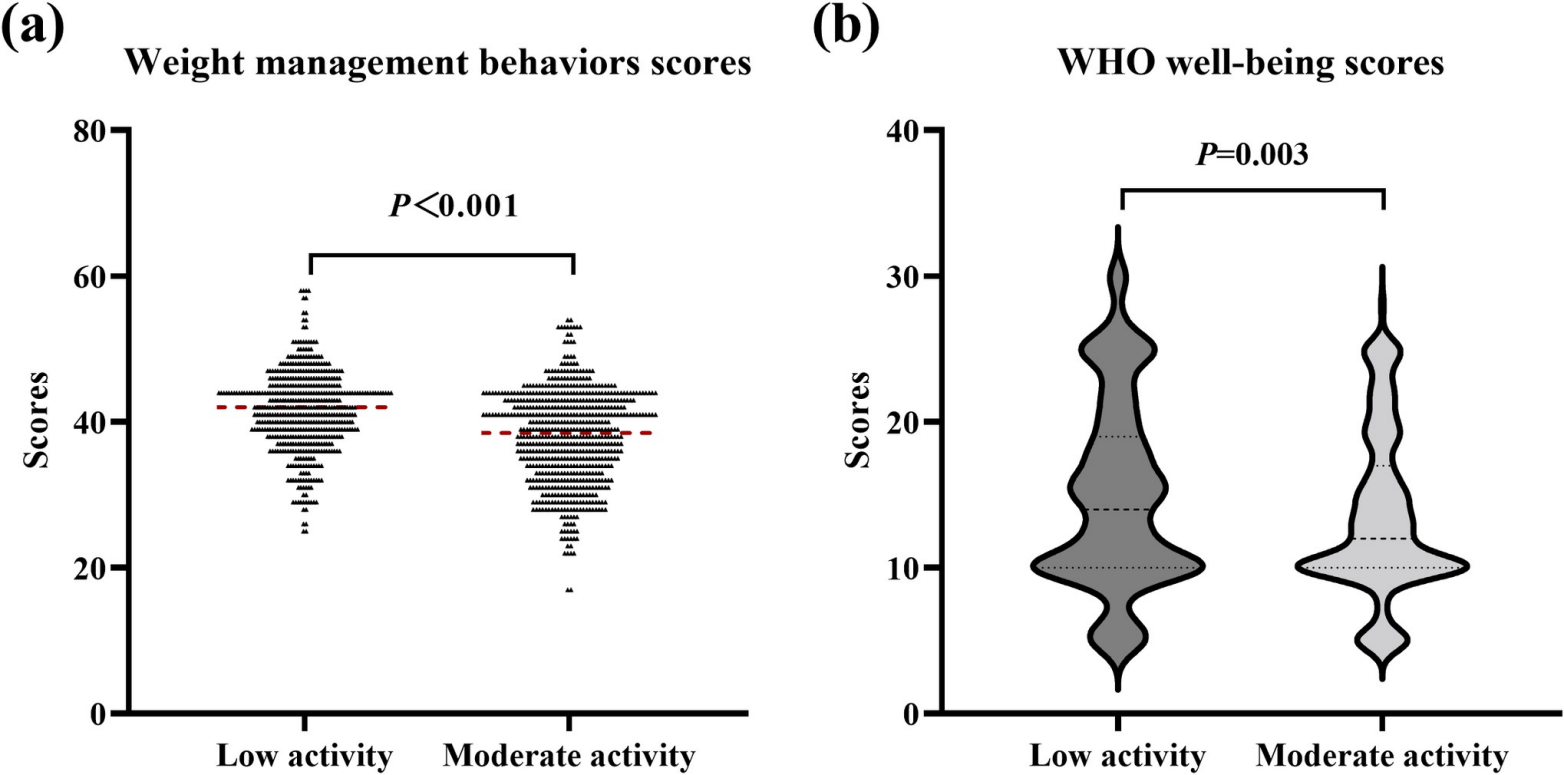
Differences in weight management behaviors and well-being scores at different levels of physical activity.

Fig 1 shows that the total weight management behavior score was significantly higher in the moderate-level PA group than in the low-level PA group (*P* < 0.001). The WHO-5 scale scores in the low-level PA group were significantly higher than those in the intermediate-level group (*P* = 0.003).

### Associations between different levels of physical activity and demographic characteristics, weight management behaviors, and well-being

The results of binary logistic regression in Table 4 showed that grade, physical activity during the non-lockdown period, weight management behavior, and well-being level had significant predictive effects on the physical activity level of healthcare students.

**Table 4:** Regression analysis of influencing factors of physical activity level.

Factors		B	S.E.	Wald	OR (95%CI)	*P*
Academic year						
	First	Ref				
	Second	-0.080	0.185	0.188	0.92(0.64–1.33)	0.664
	Third	-0.313	0.183	2.929	0.73(0.51–1.04)	0.087
	Fourth and fifth	-0.404	0.158	6.526	0.67(0.49–0.91)	0.011*
Exercise (Non-lockdown period) (No)		0.598	0.1129	21.599	1.81(1.41–2.34)	<0.001**
BMI (kg/m^2^)						
	Underweight	Ref				
	Normal	-0.237	0.165	2.060	0.80(0.58–1.09)	0.179
	Overweight or Obese	-0.057	0.208	0.075	0.95(0.63–1.42)	0.988
Weight management behaviors		-0.081	0.011	49.546	0.92(0.90–0.94)	<0.001**
WHO well-being score		-0.023	0.011	4.644	0.98(0.96–0.99)	0.031*

Notes: Ref: Reference group

* p < 0.05

** p < 0.01; B = regression coefficient and intercept; SE = standard error; OR = odds ratio; p = p-value.

## Discussion

This study aimed to understand the status quo between physical activity, weight management behaviors, and subjective well-being of healthcare students who stayed on campus during COVID-19 to attend online courses. At the same time, the influence of weight management behavior and emotional well-being on physical activity was discussed.

A recent study has found that although it is generally recommended to maintain physical exercise during the pandemic, social isolation has dramatically reduced the PA level of male and female students [36]. Data reported by the WHO shows that one in four (23%) adults need to meet global physical activity recommendations. Among the healthcare students in the six universities we included in Gansu Province, 45% had low activity levels (<600 MET minutes per week), significantly higher than the World Health Organization data. With all classroom-based classes moving online and the lack of access to university athletic facilities during school closures, it may disincentive for college students to maintain their physical activity routine and participate in regular exercise or sports [37, 38]. In addition, other measures taken during the online learning period can significantly reduce their opportunities to participate in daily physical activities, including the closure of all recreational and leisure venues, social distancing, and domestic travel restrictions. Another reason is that this may deprive them of walking time due to the cancellation of classroom sessions, resulting in a dramatic drop in physical activity levels [39–41].

Since nearly 90% of human behaviors are based on habits, daily physical activities are predominantly dominated by habits [42]. Studies have shown that when individuals have strong habits, even if their intentions are temporarily weakened, they may act according to these habits, which is conducive to maintaining behavior over time [43]. Our study found that over half of the healthcare students did not exercise during the non-blockade period. Healthcare students with exercise habits during the blockade had higher physical activity levels. An Australian randomized controlled trial found that physical activity habits in the workplace could be enhanced through habit-based interventions [44]. A combination of internal process factors influences the development of physical activity habits (e.g., cue triggering, behavior repetition, and behavior maintenance) and external environmental factors (e.g., family, school, and society) [45]. During school, we can promote participation in physical activities and the formation of fitness habits by improving the availability and convenience of school sports venues, equipment, and facilities [46]. In addition, teachers can provide the basis for teenagers to learn, imitate, and standardize, which is the critical guiding factor for establishing correct sports values and forming healthy behavior habits [47]. At the same time, peer support provides external motivation for developing good physical activity habits [48].

In addition, weight management may struggle during the COVID-19 pandemic. Higher weight management behavior scores mean poorer weight management behavior. This study found that weight management behavior scores were a potential risk factor for lower physical activity levels. Higher weight management behavior scores were associated with poorer physical activity levels. The items that scored highest on the weight management behavior Scale were used weight control products (e.g., meal replacements). An Iranian study found that students’ most common weight management methods are exercise and diet [49]. Nevertheless, students have been less physically active because of the coronavirus lockdown. At the same time, our study also found that sedentary and snacking individuals scored highly on weight management behaviors. A survey of final-year medical students in Turkey also found that before COVID-19, 16.8% of students regularly snacked, while during COVID-19, the proportion increased by 38.2% [50]. Snacking and sedentary behaviors increase obesity risk. A study of U.S. adults found that 37.2% of participants reported gaining weight during the outbreak lockdown [20]. Besides, a survey of 1013 youth in Malaysia found that 48.6% had gained weight due to the epidemic embargo [21]. In our study, 17.8% of healthcare students were overweight or obese. Some students may use weight management products to cope with weight gain due to physical inactivity, increased sedentary behavior, and snacking. Steps should be taken to inform students about positive and negative health management behaviors. This will not only help promote their health behaviors but also help guide patients in the future.

A prospective cohort study found that the number of days people participated in moderate-intensity physical activity was positively correlated with subjective well-being. However, the time spent on high-intensity physical activities was negatively associated with personal well-being [51]. Interestingly, our study found that during the COVID-19 epidemic blockade period, healthcare students with higher happiness scores had lower physical activity levels. On the one hand, the reason may be that online classes within a certain period reduce the short-term learning pressure of medical students and increase the rest time. A study on Chinese medical students shows that over 90% of undergraduates have experienced excessive sleepiness in class [52]. A survey in Lithuania found that medical students had the highest prevalence of poor sleep compared with other student groups [53]. The temporary cancellation of classroom teaching allows students to have more free time. Compared with physical activities, rest and entertainment may make them feel happier. According to the research of Bolatov et al., students become more independent and choose the time and conditions of learning during online classes, which may positively impact mental health [54]. However, it is well known that physical activity or exercise is essential to maintain physical and mental health and improve quality of life [55, 56]. Our study also shows that the physical activity level of medical students is not ideal. Therefore, guiding students on the importance of physical activities during the epidemic blockade period is very important. We should help students realize the benefits of more physical activities so that students can spend more time on physical activities in their spare time. This is very important to maintain students’ long-term physical and mental health.

Our results also found differences in physical activity levels by grade. Students in their final year had lower physical activity levels than their first-year students and spent less time in physical activity. Consistent with our findings, several studies have found that graduates are less physically active and more sedentary due to increased workloads [57]. The lockdown of the COVID-19 pandemic has temporarily halted final-year clinical placements for healthcare students, who stay on campus to spend most of their time looking for jobs. Another possible factor may be that the brief lockdown has given students more free time and that rest and recreation make them feel happier than physical activity.

## Strength and limitation

As far as we know, there is limited research on physical activity and the well-being of medical students during the epidemic blockade. This study provides information on the impact of the COVID-19 epidemic blockade on this young group’s physical activity and mental health. It identifies the influencing factors related to physical activity.

This study has some limitations. First, due to the demographic characteristics of the sample, we surveyed six schools with medical-related majors in Gansu Province, which may limit generalization to other regions. Another limitation is that due to the limitation of the epidemic at that time, our survey could not adopt the method of random sampling. Despite these limitations, these findings differ from the results of previous studies. In the future, we need to select a broad sample of data to analyze whether there are differences in the physical activity and mental health of healthcare students in different regions. Second, we have yet to collect the physical activity status of this population before the COVID-19 blockade. Future studies should compare this difference in detail.

## Conclusions

During the lockdown caused by the COVID-19 pandemic, most healthcare students in Gansu province were inactive and had low fitness levels. Significant correlates of low levels of physical activity were grade, exercise habits during the off-lockdown period, level of weight management, and well-being. The study showed that with daily online classes under the COVID-19 pandemic lockdown, they had lower levels of physical activity and some poor health management behaviors. Schools can encourage students to exercise in their free time to increase physical activity. It is necessary to encourage medical students to engage in physical activity to combat the adverse effects of COVID-19 on the mind and body. Because the Health Care Student Union will be involved in clinical work in the future, it should also set an example for its future patients. In the post-COVID-19 era, universities with specialized healthcare education can take steps to improve students’ well-being and educational needs. Such improvements could have long-term benefits for healthcare students and society.

## References

[1] Li M, Wang Q, Shen J. The Impact of Physical Activity on Mental Health during COVID-19 Pandemic in China: A Systematic Review. Int J Environ Res Public Health 2022, 19(11):6584. doi: 10.3390/ijerph19116584 . 35682172 PMC9180501

[2] Harrison E, Monroe-Lord L, Carson AD, Jean-Baptiste AM, Phoenix J, Jackson P, et al. COVID-19 pandemic-related changes in wellness behavior among older Americans. BMC Public Health 2021, 21(1):755. doi: 10.1186/s12889-021-10825-6 . 33874931 PMC8054850

[3] Javed B, Sarwer A, Soto EB, Mashwani ZU. The coronavirus (COVID-19) pandemic’s impact on mental health. Int J Health Plann Manage 2020, 35(5):993–996. doi: 10.1002/hpm.3008 . 32567725 PMC7361582

[4] Brooks SK, Webster RK, Smith LE, Woodland L, Wessely S, Greenberg N, et al. The psychological impact of quarantine and how to reduce it: rapid review of the evidence. Lancet 2020, 395(10227):912–920. doi: 10.1016/S0140-6736(20)30460-8 . 32112714 PMC7158942

[5] Gadi N, Saleh S, Johnson JA, Trinidade A. The impact of the COVID-19 pandemic on the lifestyle and behaviours, mental health and education of students studying healthcare-related courses at a British university. BMC Med Educ 2022, 22(1):115. doi: 10.1186/s12909-022-03179-z . 35189863 PMC8860134

[6] Xiang MQ, Tan XM, Sun J, Yang HY, Zhao XP, Liu L, et al. Relationship of Physical Activity with Anxiety and Depression Symptoms in Chinese College Students During the COVID-19 Outbreak. Front Psychol 2020, 11:582436. doi: 10.3389/fpsyg.2020.582436 . 33329238 PMC7714784

[7] Ma Z, Zhao J, Li Y, Chen D, Wang T, Zhang Z, et al. Mental health problems and correlates among 746 217 college students during the coronavirus disease 2019 outbreak in China. Epidemiol Psychiatr Sci 2020, 29:e181. doi: 10.1017/S2045796020000931 . 33185174 PMC7681173

[8] Hoang TD, Colebunders R, Fodjo JNS, Nguyen NPT, Tran TD, Vo TV. Well-Being of Healthcare Workers and the General Public during the COVID-19 Pandemic in Vietnam: An Online Survey. Int J Environ Res Public Health. 2021, 29;18(9):4737. doi: 10.3390/ijerph18094737 . 33946768 PMC8125447

[9] Zeng W, Chen R, Wang X, Zhang Q, Deng W. Prevalence of mental health problems among medical students in China: A meta-analysis. Medicine (Baltimore). 2019, 98(18):e15337. doi: 10.1097/MD.0000000000015337 . 31045774 PMC6504335

[10] Gallagher RP, Weaver W, Assistant GR, et al. National survey of counseling center directors. International Association of Counseling Service 2012.

[11] Dahlin M, Joneborg N, Runeson B. Stress and depression among medical students: a cross-sectional study. Med Educ 2005, 39(6):594–604. 10.1111/j.1365-2929.2005.02176.x. . 15910436

[12] Herbert C. Enhancing Mental Health, Well-Being and Active Lifestyles of University Students by Means of Physical Activity and Exercise Research Programs. Front Public Health 2022, 10:849093. doi: 10.3389/fpubh.2022.849093 . 35548074 PMC9082407

[13] Herbert C, Meixner F, Wiebking C, Gilg V. Regular Physical Activity, Short-Term Exercise, Mental Health, and Well-Being Among University Students: The Results of an Online and a Laboratory Study. Front Psychol 2020, 11:509. doi: 10.3389/fpsyg.2020.00509 . 32528333 PMC7264390

[14] World Health Organization. Global Recommendations on Physical Activity for Health; World Health Organization: Geneva, Switzerland, 2010; Volume 60.

[15] Wachira LJ, Hayker SO, Larouche R, Oyeyemi AL, Prista A, Owino GE, et al. Physical activity and active transportation behaviour among rural, peri-urban and urban children in Kenya, Mozambique and Nigeria: The PAAT Study. PLoS One 2022, 17(1):e0262768. doi: 10.1371/journal.pone.0262768 . 35061821 PMC8782337

[16] White PH, McManus M. Investing in the Health and Well-Being of Young Adults. J Adolesc Health 2015, 57(1):126. doi: 10.1016/j.jadohealth.2015.04.005 . 26095413

[17] Vankim NA, Nelson TF. Vigorous physical activity, mental health, perceived stress, and socializing among college students. Am J Health Promot 2013, 28(1):7–15. doi: 10.4278/ajhp.111101-QUAN-395 . 23470187 PMC3758412

[18] Blake H, Stanulewicz N, Mcgill F. Predictors of physical activity and barriers to exercise in nursing and medical students. J Adv Nurs 2017, 73(4):917–929. doi: 10.1111/jan.13181 . 27731886

[19] Luciano F, Cenacchi V, Vegro V, Pavei G. COVID-19 lockdown: Physical activity, sedentary behaviour and sleep in Italian medicine students. Eur J Sport Sci 2021, 21(10):1459–1468. doi: 10.1080/17461391.2020.1842910 . 33108970

[20] Bhutani S, vanDellen MR, Cooper JA. Longitudinal Weight Gain and Related Risk Behaviors during the COVID-19 Pandemic in Adults in the US. Nutrients 2021, 13(2):671. doi: 10.3390/nu13020671 . 33669622 PMC7922943

[21] Tan ST, Tan CX, Tan SS. Trajectories of Food Choice Motives and Weight Status of Malaysian Youths during the COVID-19 Pandemic. Nutrients 2021, 13(11):3752. doi: 10.3390/nu13113752 . 34836008 PMC8620045

[22] Zeigler Z. COVID-19 Self-quarantine and Weight Gain Risk Factors in Adults. Curr Obes Rep 2021, 10(3):423–433. Epub 2021 Jul 12. doi: 10.1007/s13679-021-00449-7 . 34251647 PMC8273568

[23] Kosendiak AA, Wysocki M, Krysiński P, Kuźnik Z, Adamczak B. Impact of the COVID-19 pandemic on physical activity, smoking, alcohol use, and mental well-being-A longitudinal study of nursing students at Wroclaw Medical University in Poland. Front Public Health 2023, 16;11:1249509. doi: 10.3389/fpubh.2023.1249509 . 38035301 PMC10687417

[24] Mandic D, Bjegovic-Mikanovic V, Vukovic D, Djikanovic B, Stamenkovic Z, Lalic NM. Successful promotion of physical activity among students of medicine through motivational interview and Web-based intervention. PeerJ 2020, 8:e9495. doi: 10.7717/peerj.9495 . 32714663 PMC7353914

[25] Özden G, Parlar Kiliç S. The effect of social isolation during COVID-19 pandemic on nutrition and exercise behaviors of nursing students. Ecol Food Nutr 2021, 60:663–81. doi: 10.1080/03670244.2021.1875456 . 33475005

[26] Chen C, Lu FC; Department of Disease Control Ministry of Health, PR China. The guidelines for prevention and control of overweight and obesity in Chinese adults. Biomed Environ Sci. 2004;17 Suppl:1–36. . 15807475

[27] Macfarlane DJ, Lee CC, Ho EY, Chan KL, Chan DT. Reliability and validity of the Chinese version of IPAQ (short, last 7 days). J Sci Med Sport 2007, 10(1):45–51. doi: 10.1016/j.jsams.2006.05.003 . 16807105

[28] Qu NN, Li KJ Study on the reliability and validity of international physical activity questionnaire (Chinese Vision.IPAO). Chin J Epidemiol 2004, 03:87–90. (In Chinese) 15200945

[29] Zhang X, Zhu W, Kang S, Qiu L, Lu Z, Sun Y. Association between Physical Activity and Mood States of Children and Adolescents in Social Isolation during the COVID-19 Epidemic. Int J Environ Res Public Health 2020, 17(20):7666. doi: 10.3390/ijerph17207666 . 33096659 PMC7589310

[30] von Elm E, Altman DG, Egger M, Pocock SJ, Gøtzsche PC, Vandenbroucke JP; STROBE Initiative. The Strengthening the Reporting of Observational Studies in Epidemiology (STROBE) statement: guidelines for reporting observational studies. Lancet 2007, 370(9596):1453–7. doi: 10.1016/S0140-6736(07)61602-X . 18064739

[31] Robinson E, Boyland E, Chisholm A, Harrold J, Maloney NG, Marty L, et al. Obesity, eating behavior and physical activity during COVID-19 lockdown: A study of UK adults. Appetite 2021, 156:104853. doi: 10.1016/j.appet.2020.104853 . 33038479 PMC7540284

[32] Topp CW, Østergaard SD, Søndergaard S, Bech P. The WHO-5 Well-Being Index: a systematic review of the literature. Psychother Psychosom 2015, 84(3):167–76. doi: 10.1159/000376585 . 25831962

[33] Fung SF, Kong CYW, Liu YM, Huang Q, Xiong Z, Jiang Z, et al. Validity and Psychometric Evaluation of the Chinese Version of the 5-Item WHO Well-Being Index. Front Public Health 2022, 30;10:872436. doi: 10.3389/fpubh.2022.872436 . 35433612 PMC9005828

[34] Lippi G, Henry BM, Sanchis-Gomar F. Physical inactivity and cardiovascular disease at the time of coronavirus disease 2019 (COVID-19). Eur J Prev Cardiol 2020, 27(9):906–908. doi: 10.1177/2047487320916823 . 32270698 PMC7717305

[35] Goodyear MD, Krleza-Jeric K, Lemmens T. The Declaration of Helsinki. BMJ 2007, 29;335(7621):624–5. doi: 10.1136/bmj.39339.610000.BE . 17901471 PMC1995496

[36] Schrempft S, Jackowska M, Hamer M, Steptoe A. Associations between social isolation, loneliness, and objective physical activity in older men and women. BMC Public Health 2019, 19(1):74. doi: 10.1186/s12889-019-6424-y . 30651092 PMC6335852

[37] Araújo FJO, de Lima LSA, Cidade PIM, Nobre CB, Neto MLR. Impact Of Sars-Cov-2 And Its Reverberation In Global Higher Education And Mental Health. Psychiatry Res 2020, 288:112977. doi: 10.1016/j.psychres.2020.112977 . 32302818 PMC7152919

[38] Meiring RM, Gusso S, McCullough E, Bradnam L. The Effect of the COVID-19 Pandemic Movement Restrictions on Self-Reported Physical Activity and Health in New Zealand: A Cross-Sectional Survey. Int J Environ Res Public Health 2021, 18(4):1719. doi: 10.3390/ijerph18041719 . 33578964 PMC7916664

[39] Cheng YW, Chen CF, Cheng SC, Hu FY, Huang KE, Lei TL, et al. COVID-19: Guidelines for Social Distancing. Available online: https://www.cdc.gov.tw/En/File/Get/reB429_3fV4GulfumH9Vcg (accessed on 11 February 2022).

[40] Gallè F, Sabella EA, Ferracuti S, De Giglio O, Caggiano G, Protano C, et al. Sedentary Behaviors and Physical Activity of Italian Undergraduate Students during Lockdown at the Time of CoViD-19 Pandemic. Int J Environ Res Public Health 2020, 17(17):6171. doi: 10.3390/ijerph17176171 . 32854414 PMC7504707

[41] Barkley JE, Lepp A, Glickman E, Farnell G, Beiting J, Wiet R, et al. The Acute Effects of the COVID-19 Pandemic on Physical Activity and Sedentary Behavior in University Students and Employees. Int J Exerc Sci 2020, 13(5):1326–1339. . 33042377 10.70252/QCVG2516 PMC7523895

[42] Rhodes R E, Rebar A L. (2018). Physical activity habit: complexities and controversies [M]// VERPLANKENB. The psychology of habit. Switzerland: Springer Cham, 91–109.

[43] Gardner B, Lally P, & Rebar AL. Does habit weaken the relationship between intention and behaviour? Revisiting the habit-intention interaction hypothesis. Social and Personality Psychology Compass. 2020,14(8), e12553.

[44] Hamilton K, Fraser E, Hannan T. Habit-based workplace physical activity intervention: a pilot study. Occup Med (Lond) 2019, 69(7):471–474. doi: 10.1093/occmed/kqz119 . 31504848

[45] Yan JF, Sun H, Zhang J, Liu ZD. Analysis and Implications of International Experience on the Factors, Measurement Methods and Intervention Strategies of Forming Physical Activity Habits. Journal of Beijing University of Sports. 2020,45 (04), 63–77. 10.19582/j.cnki.11-3785/g8.2022.04.006.

[46] Raynor DA, Coleman KJ, Epstein LH. Effects of proximity on the choice to be physically active or sedentary. Res Q Exerc Sport 1998, 69(1):99–103. 10.1080/02701367.1998.10607674. . 9532630

[47] Masse LC. Understanding the mechanism of physical activity behavior change: Challenges and a call for action. Psychology of Sport and Exercise, 2011,12(1):5.

[48] Hsu YW, Chou CP, Nguyen-Rodriguez ST, McClain AD, Belcher BR, Spruijt-Metz D. Influences of social support, perceived barriers, and negative meanings of physical activity on physical activity in middle school students. J Phys Act Health 2011, 8(2):210–9. doi: 10.1123/jpah.8.2.210 . 21415448 PMC8098645

[49] Saghafi-Asl M, Aliasgharzadeh S, Asghari-Jafarabadi M. Correction: Factors influencing weight management behavior among college students: An application of the Health Belief Model. PLoS One. 2021 May 20;16(5): e0252258. doi: 10.1371/journal.pone.0252258 Erratum for: PLoS One 2020, 15(2): e0228058. . 32032376 PMC7006943

[50] Bosi Bağcı TA, Kanadıkırık A, Somyürek E, Gerçek G, Tanrıkulu HB, Öntaş E, et al. Impact of COVID-19 on eating habits, sleeping behaviour and physical activity status of final-year medical students in Ankara, Turkey. Public Health Nutr 2021, 24(18):6369–6376. doi: 10.1017/S1368980021003906 . 34496994 PMC8505814

[51] Wicker P, Frick B. The relationship between intensity and duration of physical activity and subjective well-being. Eur J Public Health 2015, 25(5):868–72. doi: 10.1093/eurpub/ckv131 . 26142405

[52] Lu J, Fang GE, Shen SJ, Wang Y, Sun Q. A Questionnaire survey on sleeping in class phenomenon among Chinese medical undergraduates. Med Teach 2011, 33(6):508. . 21755615

[53] Preišegolavičiūtė E, Leskauskas D, Adomaitienė V. Associations of quality of sleep with lifestyle factors and profile of studies among Lithuanian students. Medicina (Kaunas) 2010, 46(7):482–9. . 20966622

[54] Bolatov AK, Seisembekov TZ, Askarova AZ, Baikanova RK, Smailova DS, Fabbro E. Online-Learning due to COVID-19 Improved Mental Health Among Medical Students. Med Sci Educ 2020, 31(1):183–192. doi: 10.1007/s40670-020-01165-y . 33230424 PMC7673686

[55] Bird JM, Karageorghis CI, Hamer M. Relationships among behavioural regulations, physical activity, and mental health pre- and during COVID-19 UK lockdown. Psychol Sport Exerc 2021, 55:101945. doi: 10.1016/j.psychsport.2021.101945 . 34518758 PMC8425532

[56] Vancini RL, Andrade MS, Viana RB, Nikolaidis PT, Knechtle B, Campanharo CRV, et al. Physical exercise and COVID-19 pandemic in PubMed: Two months of dynamics and one year of original scientific production. Sports Med Health Sci 2021, 3(2):80–92. doi: 10.1016/j.smhs.2021.04.004 . 34189482 PMC8105136

[57] Castro O, Bennie J, Vergeer I, Bosselut G, Biddle SJH. How Sedentary Are University Students? A Systematic Review and Meta-Analysis. Prev Sci 2020, 21(3):332–343. doi: 10.1007/s11121-020-01093-8 . 31975312

